# Does serum neurofilament light help predict accelerated cognitive ageing in unimpaired older adults?

**DOI:** 10.3389/fnins.2023.1237284

**Published:** 2023-08-10

**Authors:** Jessica M. Collins, Aidan D. Bindoff, Eddy Roccati, Jane E. Alty, James C. Vickers, Anna E. King

**Affiliations:** ^1^Wicking Dementia Research and Education Centre, University of Tasmania, Hobart, TAS, Australia; ^2^School of Medicine, University of Tasmania, Hobart, TAS, Australia; ^3^Royal Hobart Hospital, Hobart, TAS, Australia

**Keywords:** biomarkers, neurofilament light, cognitive ageing, cognition, mediation analysis

## Abstract

**Introduction:**

Neurofilament light (NfL) is a blood biomarker of neurodegeneration. While serum NfL levels have been demonstrated to increase with normal ageing, the relationship between serum NfL levels and normal age-related changes in cognitive functions is less well understood.

**Methods:**

The current study investigated whether cross-sectional serum NfL levels measured by single molecule array technology (Simoa®) mediated the effect of age on cognition, measured by a battery of neuropsychological tests administered biannually for 8 years, in a cohort of 174 unimpaired older adults (≥50 years) from the Tasmanian Healthy Brain Project. Mediation analysis was conducted using latent variables representing cognitive test performance on three cognitive domains - episodic memory, executive function, and language (vocabulary, comprehension, naming). Cognitive test scores for the three domains were estimated for each participant, coincident with blood collection in 2018 using linear Bayesian hierarchical models.

**Results:**

Higher serum NfL levels were significantly positively associated with age (*p* < 0.001 for all domains). Cognitive test scores were significantly negatively associated with age across the domains of executive function (*p* < 0.001), episodic memory (*p* < 0.001) and language (*p* < 0.05). However, serum NfL levels did not significantly mediate the relationship between age and cognitive test scores across any of the domains.

**Discussion:**

This study adds to the literature on the relationship between serum NfL levels and cognition in unimpaired older adults and suggests that serum NfL is not a pre-clinical biomarker of ensuing cognitive decline in unimpaired older adults.

## 1. Introduction

Cognitive decline with ageing is common; however, the onset, extent and rate of cognitive decline is highly variable between individuals (Rowe and Kahn, 1987; Nyberg et al., 2020). Age-related cognitive decline can range from minimal decline, which can be viewed as successful ageing through to levels of decline that meet criteria for mild cognitive impairment (MCI) and dementia (Rowe and Kahn, 1987; Nyberg et al., 2020). Cognitive impairment below the threshold of MCI and dementia is associated with a reduced quality of life (Roehr et al., 2017), and for some people may be a pre-clinical phase, on the spectrum of accelerated cognitive decline, continuous with dementia (Jessen et al., 2014). Being able to sensitively detect who has accelerated cognitive ageing from normal cognitive ageing, will enable the early identification of people at risk of impairment, hence providing an opportunity for targeted dementia prevention.

Cognitive decline is due to age- and pathology-related neurodegenerative changes in the brain, which precede clinical cognitive changes by several years (Fleisher et al., 2015; Quiroz et al., 2018). Several blood-based biomarkers of these neurodegenerative changes in the brain have now been identified and are the subject of ongoing investigation (Alcolea et al., 2023). One such biomarker is the neuroaxonal protein neurofilament light (NfL), which is particularly abundant in a subset of neurons, typically projecting neurons, with wide calibre, myelinated axons (Kirkcaldie and Dwyer, 2017).

Increased serum and plasma levels of the NfL protein have been demonstrated in several neurodegenerative diseases including Alzheimer’s disease (AD) (Lewczuk et al., 2018; Forgrave et al., 2019; Moscoso et al., 2021), frontotemporal dementia (Forgrave et al., 2019), motor neuron disease (Forgrave et al., 2019) and traumatic brain injury (Shahim et al., 2018). Thus, NfL does not appear to be a specific biomarker, for a particular neurological disorder, but is a general marker of neurodegenerative processes, particularly if they involve axonal pathology. Whilst NfL concentrations are slightly higher when measured in serum samples compared to plasma samples, the difference is minimal and NfL levels between the two sample types are highly correlated (Barro et al., 2020). Therefore, although the sample type may be important in the determination of reference ranges and diagnostic cut-off values, the associations between NfL levels, and measures of cognition and brain atrophy are expected to be concordant between serum and plasma samples.

NfL levels in serum and plasma have been demonstrated to significantly increase with aging in a multitude of studies (Disanto et al., 2017; Khalil et al., 2020; Quiroz et al., 2020; Kang et al., 2021). The biological source of the age-related increase in blood NfL levels is unclear but may reflect age-related neurodegeneration in the brain (Alirezaei et al., 2020; Khalil et al., 2020; Nyberg et al., 2020; Kang et al., 2021). Longitudinal analyses have revealed marked variability in age-related NfL increases in the blood (Khalil et al., 2020; Nyberg et al., 2020) and a study in neurologically inconspicuous community-dwelling adults has shown that serum NfL levels become more variable between individuals with increasing age (Khalil et al., 2020). In the same study, baseline serum NfL levels were shown to be a predictor of future brain volume loss and NfL levels increased in relation to brain volume loss (Khalil et al., 2020). Additionally, a study by Beydoun et al. (2023) demonstrated that plasma NfL has potential as a prognostic marker of white matter integrity in a study of middle-aged urban adults (mean age 47.9 years). Therefore, the variability in age-related increases in serum NfL levels in cognitively normal individuals, may be explained by pre-clinical neurodegenerative changes in the brain, beyond that of normal ageing, but not to the extent of clinical impairment. In support of this hypothesis, blood NfL levels are associated with risk of all-cause (de Wolf et al., 2020; Verberk et al., 2021) and AD dementia (de Wolf et al., 2020; Stocker et al., 2023). Furthermore, plasma NfL levels have been demonstrated to be a predictor of brain amyloid beta (Aβ) positivity as measured by Florbetapir-PET in participants with normal cognition suggesting it is a sensitive biomarker of the preclinical AD phase (Huang et al., 2022), however the relationship between Aβ burden and AD risk remains controversial.

Whilst studies have shown a relationship between serum and plasma NfL levels and cognition in people with mild cognitive impairment (MCI) (Lewczuk et al., 2018; Mattsson et al., 2019; Osborn et al., 2019) and AD (Lewczuk et al., 2018; Mattsson et al., 2019; Quiroz et al., 2020), the relationship between serum NfL levels and pre-clinical changes in cognitive status in unimpaired adults is less understood. Most of the literature on the relationship between NfL levels in the blood and cognition has been analysed in three main ways: first, the cross-sectional relationship between NfL and cognition at a single timepoint (Mielke et al., 2019; Osborn et al., 2019; Huang et al., 2022); second, the relationship between baseline NfL levels and future cognitive decline (Khalil et al., 2020; Verberk et al., 2021; Chatterjee et al., 2022); third, the longitudinal relationship between the rate of NfL change and cognitive decline trajectories (Mattsson et al., 2019; Mielke et al., 2019; Rauchmann et al., 2021). Furthermore, studies differ on the tools used to measure cognition, ranging from single instrument measures such as Mini Mental State Exam (MMSE) (Khalil et al., 2020; Chatterjee et al., 2022) to neuropsychological test batteries (Osborn et al., 2019; Rauchmann et al., 2021; Verberk et al., 2021).

When looking at the cross-sectional relationship between blood NfL and cognition, a study by Osbourn et al. found that higher plasma NfL was not associated with performance across multiple cognitive domains in unimpaired participants between the ages of 60 and 92 years (Osborn et al., 2019). In contrast to this, a more recent cross-sectional analysis in participants over 50 years of age, with normal cognition or objectively defined subtle cognitive impairment (Obj-SCD), showed that higher NfL levels were associated with lower global cognition, verbal episodic memory, visual episodic memory and executive function (Huang et al., 2022). However, when looking at subgroup analyses based on cognition (normal and Obj-SCD) and brain amyloid beta (Aβ+ and Aβ−) status there were differing results; NfL had a negative correlation with verbal episodic memory in the Aβ− normal cognition and Aβ− Obj-SCD groups, and a negative association with MMSE in the Aβ+ normal cognition group (Huang et al., 2022). A study of participants without dementia, but including people with both normal cognition and MCI, and a mean age of 76 years of age has also found no cross-sectional relationship between plasma NfL levels and global cognition or any cognitive measures using a 9-test neuropsychological battery (Mielke et al., 2019). This demonstrates that the association between blood NfL levels and cognition, in cognitively unimpaired older adults is complex and may vary based on both brain Aβ status and whether the cohort of cognitively normal participants includes those with subjective cognitive complaints or MCI.

Analysing the association between baseline blood NfL measures and cognition, a study by Khalil et al. (2020) demonstrated that baseline serum NfL levels correlated with future changes in MMSE scores over a mean follow-up time of 5.9 years, in a population of healthy adults aged between 38 and 85 years of age, but the annualised change in serum NfL was not related to the annualised change in MMSE scores. This is supported by a more recent study showing that baseline plasma NfL levels were significantly associated with prospective cognitive decline (measured by MMSE and the Preclinical Alzheimer’s Cognitive Composite) in cognitively unimpaired participants with a mean age 74.2 (SD 7.2) years (Chatterjee et al., 2022). Conversely, Verberk and colleagues have shown that higher baseline serum NfL levels were not associated with the rate of decline across the cognitive domains of memory, attention, executive function, language and global cognition in cognitively normal participants with a mean baseline age of 61 years (Verberk et al., 2021). However, it is important to note that this study enrolled participants from a memory clinic who despite not meeting criteria for MCI or dementia, did present at the memory clinic due to cognitive complaints (Verberk et al., 2021). Therefore, there is conflicting evidence for the relationship between higher baseline NfL levels in the blood as a predictor of future cognitive decline, and this may be a result of the cognitive measures used and/or the cohort studied.

When looking at the longitudinal relationship between plasma NfL levels and cognition, a study of participants without dementia but including those with both normal cognition and MCI, and a mean age of 76 years found that change in plasma NfL levels was associated with change in attention and global cognitive scores over a 30-month follow-up period (Mielke et al., 2019). Similarly, a study of participants with normal cognition through to dementia aged between 55 and 90 years of age demonstrated that plasma NfL rate of change was predictive of a memory composite score over a 4 year follow-up (Rauchmann et al., 2021). In contrast, in a cohort including cognitively unimpaired, MCI and AD dementia participants with a mean age of 72.9 (SD 7.1) years, faster increasing plasma NfL levels correlated with a faster decline in global cognition in participants with MCI and dementia, but not in cognitively unimpaired participants (Mattsson et al., 2019). When taken together the literature demonstrates that the relationship between blood NfL levels and cognitive functions is complex in cognitively normal older adults and requires further elucidation before NfL’s clinical utility as a prognostic biomarker of cognitive decline can be determined.

The aim of the current study was to estimate the proportion of variance in cognitive functions mediated by pre-clinical neurodegeneration (measured by serum NfL), in order to gain a better understanding of the utility of serum NfL as a biomarker of pre-clinical cognitive decline. We have used longitudinal data from an extensive battery of cognitive tests in a cohort of cognitively unimpaired older adults in the Tasmanian Healthy Brain Project (THBP) to estimate cognitive functions in the same year (2018) as (cross-sectional) blood collection, on three cognitive domains (executive function, episodic memory, and language).

## 2. Methods

### 2.1. Participants

Participants were from the THBP, a longitudinal intervention study of older, community-dwelling Tasmanians which commenced in 2010 (Summers et al., 2013). Detailed information on the establishment of THBP and recruitment of its participants has been published previously (Summers et al., 2013). Briefly, adults aged between 50 and 79 years of age, without a history of prior conditions known to be associated with impaired neurological function, or neurological/psychiatric disorders were eligible. At recruitment, participants opted (non-random assignment) to undertake university-level education (completed at least 12.5% of 1 year full-time equivalent university study, later-life education intervention group) or no further education (comparison group). At baseline, participants also completed a medical health status questionnaire and data were collected on demographics using a specifically designed questionnaire including age (years), gender (options either “male” or “female”), education (in years) and occupational history (Summers et al., 2013). Education (in years) was calculated as the sum of the highest school leaving year and the number of years of further education (both determined using the baseline medical questionnaires) and the years of full-time-equivalent university study as part of the THBP (determined using academic transcripts).

All participants underwent annual cognitive assessments for the first 4 years of the study and then biennial thereafter. All THBP participants were invited to take part in the current study, with 174 (39 comparison group, 22%; 135 later-life education intervention 78%) of the remaining 410 participants at the time of collection in 2018 consenting to provide a blood sample. This project was approved by the University of Tasmania Health and Medical Research Committee (H0018265 and H0016317).

### 2.2. Cognitive assessments

Participants had comprehensive cognitive assessments between 2011 and 2022, as previously detailed (Summers et al., 2013). Briefly, a neuropsychological battery of tests was performed at each assessment, evaluating the cognitive domains of episodic memory [Paired Associates Learning Test - Cambridge Neuropsychological Test Automated Battery (CANTAB), Rey Auditory Verbal Learning Test, Rey Complex Figure Test, Wechsler Memory Scale (WMS)-III Logical Memory Test I & II], language [Wechsler Adult Intelligence Scale (WAIS)-III Vocabulary, WAIS-III Comprehension, Boston Naming Test] and executive function [Stroop Test C, Trail Making Test Part B, WAIS-III Digit Span Test, Controlled Oral Word Association Test].

Blood sample collection from consenting participants in 2018, did not necessarily coincide with a cognitive assessment, however all participants who provided blood had cognitive assessments before and after blood collection. We chose to single-impute cognitive tests scores for the year in which blood collection occurred (2018) by modelling individual trajectories on each test between 2015 and 2022 in order to make economical use of the available data using Bayesian hierarchical models (the decision process and methods are detailed in Supplementary material).

### 2.3. Blood collection and processing

The collection, processing and storage of blood was performed in 2018 according to the published guidelines for Alzheimer’s disease research (O’Bryant et al., 2015). Non-fasting blood samples were collected under aseptic conditions by venepuncture using a 21-gauge (G) butterfly needle into BD Vacutainer™ SST™ II Advance tubes (Cat no. 367958). To prepare serum, blood was clotted at room temperature in a vertical position for 30 min and then centrifuged at 1,500 g for 10 min at 4°C. Each serum sample was aliquoted into 10 polypropylene, screw-top cryostorage tubes to prevent multiple freeze–thaw cycles and stored at −80°C. This process was completed in under 2 h from venepuncture.

### 2.4. Genotyping

*APOE* genotypes were available for the THBP cohort from a previous study (Ward et al., 2014). DNA was collected from THBP participants with the Oragene DNA self-collection kit (Genotek, ON, Canada, 2012). A one-step amplified refractory mutation system PCR, followed by gel electrophoresis was used to determine *APOE* genotype. Using methods previously described (Donohoe et al., 1999), rs429358 and rs7412 were determined with PCR amplifications undertaken using ~50 ng of genomic DNA and PCR amplicons resolved on a 2% agarose gel. Samples were run in duplicate to ensure accuracy of genotyping, and genotyping repeated if results were unclear.

### 2.5. Serum NfL measurements

Serum NfL levels were measured using the NF-LIGHT™ (SR-X VERSION) single molecule array assay (SIMOA®) from Quanterix™ (cat number 103400). Serum samples were measured in duplicate using a 2-step assay on the SIMOA® SR-X™ platform (Quanterix™), according to manufacturer’s protocols. Serum samples were diluted 4 times in sample diluent by adding 25 μL of serum to 75 μL of sample diluent. High- and low- quality control samples provided by Quanterix™ were run on each plate. If the intra-assay coefficient of variation between two duplicates was more than 20%, the sample was measured again. The average intra-assay coefficient of variation for included samples was 5.77%.

### 2.6. Statistics

Mediation analysis was conducted using latent variables representing cognitive test performance on three cognitive domains - episodic memory, executive function, and language. Cognitive test scores were estimated for each participant coincident with blood collection in 2018 using the hierarchical regression models, which considered age and length of time in the study in 2018. These scores were then standardized by converting to z-scores and reversing two items so that all items were positively correlated. We determined which cognitive tests to include on each cognitive domain using published literature (Smith et al., 2013; Faria et al., 2015) and earlier work using baseline results from the THBP (Ward et al., 2017), then proceeded using an iterative confirmatory factor analysis (CFA) approach to target a comparative fit index (CFI) > 0.95, and root mean squared error of approximation (RMSEA) < 0.05. If a model did not satisfy these criteria, we inspected the correlation matrix to identify items (cognitive tests) which were considered either redundant (highly correlated with another item) or poorly correlated with other items. The cognitive tests included for each cognitive domain, their loadings and 95% CIs are included in Table 1.

**Table 1:** Cognitive domain latent variables, cognitive tests comprising them and factor loadings ± 95% CI.

Cognitive domain	Cognitive tests in model	LV loading	Lower CI of LV loading	Upper CI of LV loading	CFI	RMSEA	Model test statistic	value *p*	*R* ^2^
Executive Function	Trail making test part B	0.875	0.749	1.001	0.939	0.116	$\chi_{7}^{2}=20.6$	0.004	0.552
	Digit-span, WAIS-III	0.473	0.330	0.616					
	Stroop C	0.657	0.515	0.799					
Episodic Memory	Paired associates learning	0.707	0.604	0.811	0.995	0.033	$\chi_{7}^{2}=8.09$	0.325	0.367
	Rey complex figure test	0.877	0.782	0.973					
	Logical memory (I) subtest, WME	0.536	0.363	0.709					
Language	Boston naming test	0.481	0.291	0.671	0.928	0.109	$\chi_{7}^{2}=18.82$	0.009	0.329
	Vocabulary, WAIS-III	0.782	0.598	0.967					
	Comprehension, WAIS-III	0.643	0.464	0.882					

LV, latent variable; CI, confidence interval; CFI, comparative fit index; RMSEA, root mean squared error of approximation; WAIS-III, Wechsler Adult Intelligence Scale-Third; WME, Wechsler Memory Scale.

**Figure 1:**
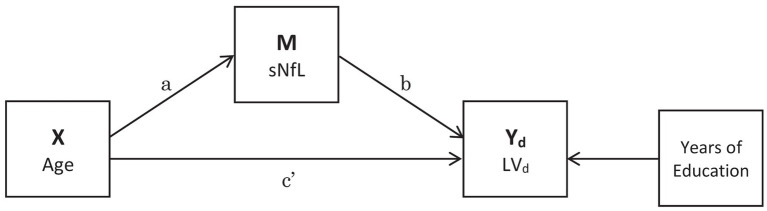
Mediation analysis pathways. The indirect pathway is a*b, the direct pathway is c’ and the proportion mediated is a*b/(a*b + c’). Years of Education was included as a covariate. Latent variable (LV).

The X → M → Y_d_ ← X paths were estimated using multiple linear regression, where X was participant age (mean-centered and standardized to unit SD), M was serum NfL concentration (log_e_-transformed due to right-skew), Y_d_ were the latent variable scores on cognitive domain *d* and years of education was included as a covariate (Figure 1). This model proposes that age causes both cognitive decline and neurodegeneration (measured using the proxy of NfL in serum), and that neurodegeneration is a mediator of age-related cognitive decline, evident in serum NfL concentrations.

We used the product of coefficients method to estimate the indirect a*b path (X → M → Y_d_), direct path c’ (Y_d_ ← X) and proportion mediated (a*b/(a*b + c’); see Figure 1). Standard errors were computed using bootstrapping (1 × 10^3^ iterations). We considered a path coefficient to be significant at α = 0.05. To avoid complexity we fitted a SEM for each cognitive domain rather than estimating all latent variables simultaneously. SEM was conducted using the ‘lavaan’ R package (Rosseel, 2012).

## 3. Results

Blood samples from *N* = 174 participants were analysed. Mean serum NfL concentration was 14.3 pg/mL (SD = 6.6). Some participants had insufficient cognitive assessment data to estimate cognitive domain scores, leaving *n* = 143 participants who undertook 528 cognitive assessments between 2015 and 2022. Mean age of participants was 65.5 years (SD = 6.8), 71.3% were female. Mean years of education was 17.1 years (SD = 3.2). The results of the CFA iterative approach for the cognitive domain latent variables, executive function, episodic memory, and language are included in Table 1 (further details on the CFA are included in Supplementary material).

### 3.1. Mediation

**Figure 2:**
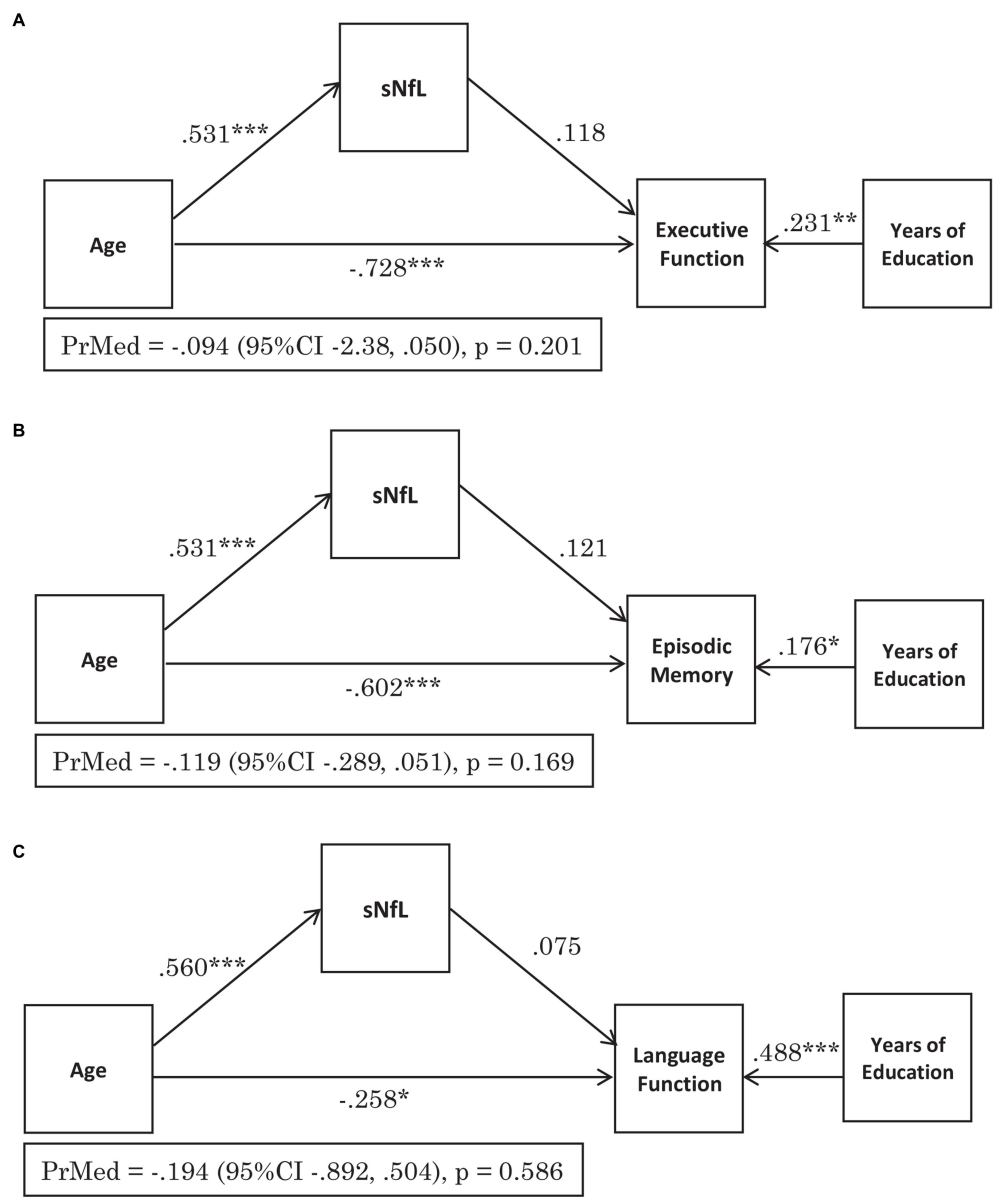
Serum NfL does not mediate the effect of age on cognitive function across the domains of: **(A)** Executive Function, **(B)** Episodic Memory and **(C)** Language. The figures demonstrate the standardised regression coefficients (β), *p*-values for the pathways and the proportion mediated (PrMed) β values, 95% confidence intervals (95% CI) and *p*-values. ^*^p < 0.05, ^**^p < 0.01, ^***^p < 0.001.

The standardised regression coefficient β = −0.728 [95% CI −0.848, −0.607] for the effect of age on executive function, adjusted for education and serum NfL was significant (*p* < 0.001; Figure 2A). The effect of age on serum NfL had the standardised regression coefficient β = 0.531 [95% CI 0.432, 0.630] which was also significant (*p* < 0.001), however the standardized regression coefficient β = 0.118 [95% CI −0.54, 0.289] for the effect of serum NfL on executive function was not (*p* = 0.179; Figure 2A). The indirect effect standardised regression coefficient β = 0.062 [95% CI −0.031, 0.156] was also not significant (*p* = 0.190), therefore serum NfL did not mediate the effect of age on executive function.

For episodic memory, the standardised regression coefficient β = −0.602 [95% CI −0.760, −0.444] for the effect of age, adjusted for education and serum NfL was significant (*p* < 0.001), as was the standardised regression coefficient β = 0.531 [95% CI 0.431, 0.631] for the effect of age on serum NfL (*p* < 0.001; Figure 2B). The standardised regression coefficient for the effect of serum NfL on episodic memory adjusted for age β = 0.121 [95% CI −0.044, 0.286] was not significant (*p* = 0.151; Figure 2B). The effect of age on episodic memory score was not mediated by serum NfL levels, with an indirect effect standardised regression coefficient of β = 0.064 [95% CI −0.025, 0.153] (*p* = 0.159).

The effect of age on language, adjusted for education and serum NfL was significant (*p* < 0.05) with the standardised regression coefficient β = −0.258 [95% CI −0.469, −0.048] (Figure 2C). The standardised regression coefficient β = 0.560 [95% CI 0.449, 0.672] for the effect of age of serum NfL levels was also significant (*p* < 0.001), however the standardised regression coefficient β = 0.075 [95% CI −0.162, 0.312] for the effect of serum NfL on language, adjusted for age was not significant (*p* = 0.536, Figure 2C). There was also no mediation of serum NfL levels on the effect of age on language as the indirect effect standardised regression coefficient of β = 0.042 [95% CI −0.090, 0.174] was not significant (*p* = 0.534).

**Figure 3:**
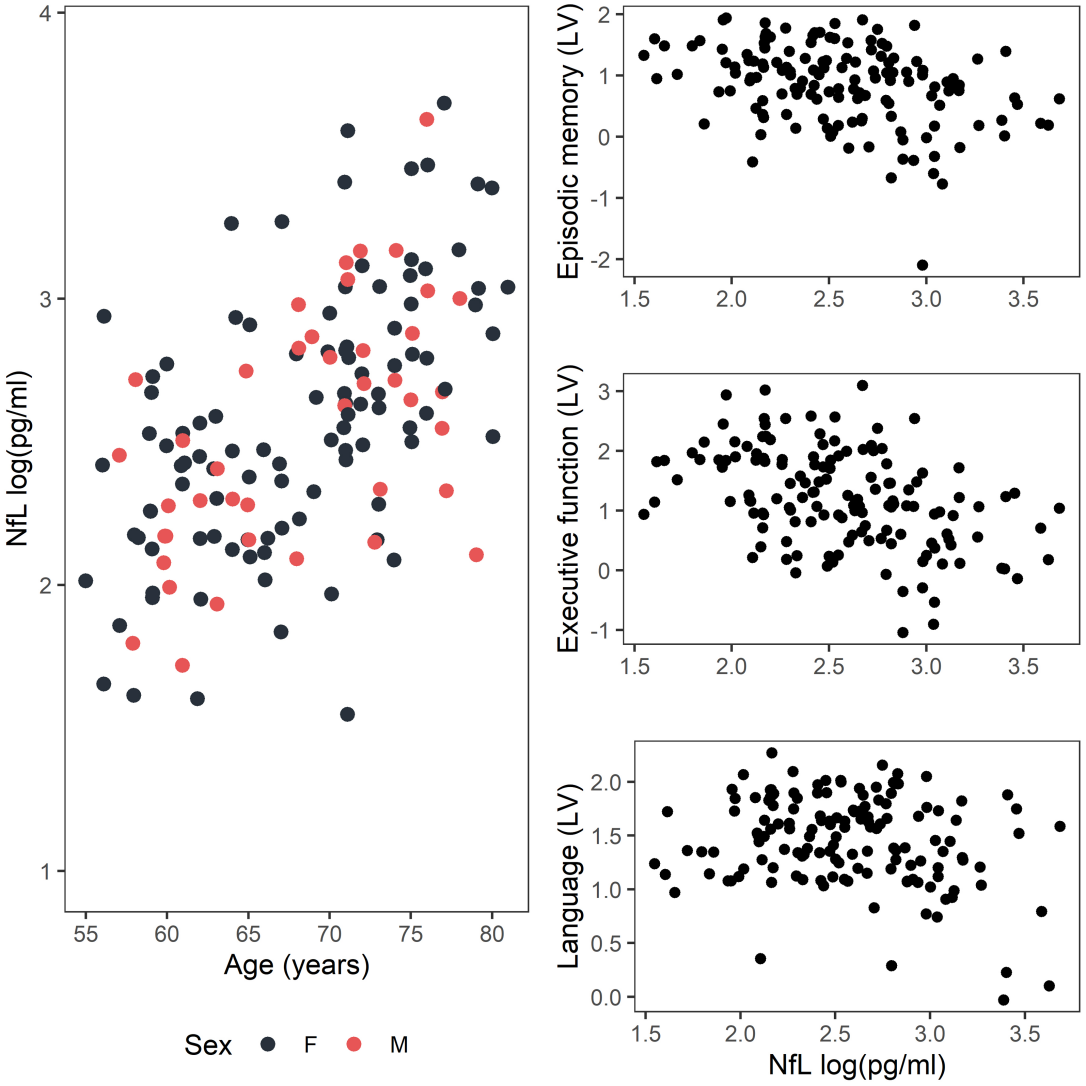
Distribution of data for the relationship between log_e_-transformed serum NfL and age for females (F, black) and males (M, red) and between log_e_-transformed serum NfL and the cognitive domain latent variables (LV) for episodic memory, executive function, and language.

The relationship between log_e_-transformed serum NfL and age and the distribution of log_e_-transformed serum NfL and the cognitive domain latent variables are shown in Figure 3. A sensitivity analysis was also performed using raw cognitive tests scores for participants who had cognitive testing coincident (in 2018) with blood collection, and the results support those of the Bayesian approach (full statistical methodology and results included in Supplementary material). Supplementary material also includes statistical methodology and results for the interactions between serum NfL and APOE genotype, age and APOE genotype and serum NfL and sex, on cognitive domain latent variables, none of which were statistically significant.

## 4. Discussion

The aim of the current study was to determine if serum NfL levels, used as a proxy measure of neurodegeneration, mediates the effects of age on cognitive functions, in unimpaired older adults. We hypothesised that in unimpaired older adults, age causes both cognitive decline and higher serum NfL levels, indicative of neurodegeneration, and that NfL levels (neurodegeneration) mediate age-related cognitive decline. This study did not detect a statistically significant mediation effect of serum NfL levels on the association between age and cognitive test scores across the domains of executive function, episodic memory or language, thereby negating this hypothesis.

The results of this study suggest that in cognitively unimpaired older adults, serum NfL levels do not provide us with any extra information on cognitive status beyond that which we can predict using chronological age. This is supported by several studies which have also demonstrated a lack of association between blood NfL levels and cognition, adjusted for age (Mielke et al., 2019; Osborn et al., 2019). It is possible that cognitive reserve compensates for the neuronal pathology/neurodegeneration indicated by blood NfL levels in unimpaired older adults and therefore no relationship between blood NfL levels and cognition is apparent. In support of this hypothesis, the current study demonstrated significant positive associations between years of education (a proxy of cognitive reserve) and cognitive tests score across all three cognitive domains.

In support of previous studies (Disanto et al., 2017; Khalil et al., 2020; Quiroz et al., 2020; Kang et al., 2021), blood NfL levels were significantly correlated with age in this cohort of unimpaired older adults. The brain is known to undergo atrophy with ageing, with white matter loss greater than grey matter loss (Harada et al., 2013). In ageing, this atrophy is attributed to decreases in neuronal size, dendritic complexity and arborisation and the number of synaptic connections, rather than overt cell death (Harada et al., 2013). As NfL is expressed throughout the dendrites, cell body and axon of neurons (Yuan et al., 2017), it is possible that NfL is released and enters the blood when these cells undergo partial or complete degeneration however, the mechanisms underlying this remain unknown. This hypothesis is supported by studies demonstrating that the increase in NfL in the blood correlates with brain volume loss on MRI (Mielke et al., 2019; Khalil et al., 2020) and reduced white matter integrity measured by DTI (Beydoun et al., 2023). Therefore, the increase in circulating NfL levels with age may be due to ageing-related neuronal damage and degeneration. However, the result of the current study and others (Mattsson et al., 2019; Osborn et al., 2019; Verberk et al., 2021) suggests that neurodegeneration indicated by blood NfL is not part of the causal relationship between aging and lower cognitive test scores in unimpaired older adults. Other factors may affect the levels of NfL in the blood, including glymphatic clearance (Plog et al., 2015), blood–brain barrier function (Uher et al., 2021) and kidney function (Akamine et al., 2020). Variability in NfL levels in the blood due to these confounding variables may be equal to or larger than the changes due to subtle, age-related or early neurodegenerative changes in the brains of unimpaired older adults. However, in MCI and dementia more extensive neurodegenerative changes in the brain may lead to NfL changes in the blood that far exceed the NfL changes caused by confounding factors such as kidney function. Therefore, the relationship between blood NfL levels and cognition may be more easily detected in MCI and dementia, hence resulting in the significant association reported for these conditions (Lewczuk et al., 2018; Mattsson et al., 2019; Osborn et al., 2019; de Wolf et al., 2020; Quiroz et al., 2020). It is also possible that the relationship between blood NfL levels and cognitive functions differs in disease compared to normal ageing and that the mechanisms of NfL release from cells to the blood are different.

In line with a substantial body of research demonstrating that cognitive test scores are significantly associated with age (Christensen, 2001; Deary et al., 2009), the current study demonstrated that higher age was associated with lower cognitive test scores across the cognitive domains of executive function, episodic memory and language. Age-related decline in cognitive test scores is likely due in part, but not solely, to neurodegeneration, with other molecular and biological factors, such as hormonal dysregulation and cognitive factors including changes in the speed/accuracy trade-off involved (Brebion, 2001; Deary et al., 2009; Bishop et al., 2010).

A novel aspect of the current study was a more thorough determination of cognitive function by using cognitive trajectories for each participant between 2015 and 2022 and estimating their cognitive test scores coincident with the 2018 serum NfL analysis. This contrasts with previous cross-sectional studies that have looked at single timepoint cognitive test scores coincident with blood NfL measures (Osborn et al., 2019; Huang et al., 2022). This study used cognitive data both preceding and succeeding the NfL analysis to determine participants cognitive test scores more accurately at the time of NfL analysis, rather than relying on a single timepoint test score.

It is important to note that in our cohort of older adults, serum NfL levels were relatively low (range 4.7–39.8 pg./mL). This may reflect the demographics of the cohort, which in comparison to the general Australian population, have more years of education, a higher IQ and likely a higher socio-economic status (Thow et al., 2018). Thus, the low serum NfL concentrations may indicate healthier ageing of this cohort. However, the range of values seen in the current study are comparable to those published previously in a cohort of cognitively unimpaired older adults (Khalil et al., 2020).

A limitation of the current study is the cross-sectional measurement of serum NfL. Recent studies have shown that the rate of change of NfL in the blood, rather than the absolute concentration may be a better indicator of ensuing cognitive decline and dementia (Mielke et al., 2019; Hadjichrysanthou et al., 2020). Future studies focusing on the longitudinal relationship between blood NfL levels and cognitive change in unimpaired older adults are planned. Furthermore, mediation analyses assume that there is no measurement error or unmeasured confounding in the mediator. This is a limitation for NfL as we know that its levels are confounded by age (Khalil et al., 2020; Koini et al., 2021), body mass index (BMI; Koini et al., 2021) and kidney function (Akamine et al., 2020). We were able to adjust for age in our mediations analyses, but not for kidney function or BMI or any other unknown confounders. Furthermore, all lab-based assays will carry some amount of measurement error. Mediation analysis was chosen due to the strong colinear relationship between age, serum NfL and cognition however, this limitation must be kept in mind when interpreting the results of this study. It also should be noted that the current study did not gather information on the presence of subjective cognitive complaints, instead focusing on objective cognitive measures. It is possible that the relationship between serum NfL and cognition is different between cognitively unimpaired people with, and without, subjective cognitive changes.

In conclusion, the current study found no mediating effect of serum NfL levels on cognitive tests scores in unimpaired older adults. This study adds to the literature investigating the relationship between blood NfL levels and cognition during aging and suggests that serum NfL levels may not be useful in the pre-clinical detection of early cognitive changes.

## Data availability statement

The original contributions presented in the study are included in the article/Supplementary material, further inquiries can be directed to the corresponding author.

## Ethics statement

The studies involving human participants were reviewed and approved by University of Tasmania Health and Medical Research Committee. The patients/participants provided their written informed consent to participate in this study.

## Author contributions

JC conducted serum NfL analysis. JC and AB conducted statistical analyses and prepared tables and figures. JC, AK, and JV conception and design of the study and funding acquisition. JC wrote the initial manuscript draft. All authors contributed to data interpretation, wrote sections of the manuscript, contributed to manuscript revisions, read and approved the submitted version of the manuscript.

## Funding

This project is funded by National Health and Medical Research Council (NHRMC) Project grants (1003645 and 1108794) and Boosting Dementia Research Leadership Fellowship (APP1136913); the JO and JR Wicking Trust (Equity Trustees); and the Royal Hobart Hospital Research Foundation (20-003).

## Conflict of interest

The authors declare that the research was conducted in the absence of any commercial or financial relationships that could be construed as a potential conflict of interest.

## Publisher’s note

All claims expressed in this article are solely those of the authors and do not necessarily represent those of their affiliated organizations, or those of the publisher, the editors and the reviewers. Any product that may be evaluated in this article, or claim that may be made by its manufacturer, is not guaranteed or endorsed by the publisher.
