# Supplementary material for: Does serum neurofilament light help predict accelerated cognitive ageing in unimpaired older adults?
Source: Front Neurosci. 2023 Aug 10;17:1237284. doi: 10.3389/fnins.2023.1237284 (PMC10448959; doi:10.3389/fnins.2023.1237284)
Supplement: Supplementary file 1 [file Data_Sheet_1.docx]

**Supplementary Materials**

**Bayesian hierarchical modelling of cognitive test scores**

Blood sample collection from consenting participants in 2018 did not necessarily coincide with a cognitive assessment, however all participants who provided blood had cognitive assessments before and after blood collection. We chose to single-impute all cognitive tests scores for the year in which blood collection occurred (2018) by modelling individual trajectories on each test between 2015 and 2022 in order to make economical use of the available data and avoid potential biases resulting from the use of simple decision rules for imputation of missing cognitive test scores. For example, had we chosen "last observation carried forward" or "next observation carried backward" then we would lose information from cognitive assessments after/before blood collection, and the time between these observations (cognitive assessment and blood collection) would have varied between participants.

We fitted linear Bayesian hierarchical models to estimate participant-level cognitive trajectories on each test in a battery of cognitive tests (tests are detailed in Methods ‘2.2 Cognitive assessments’). By-participant correlated random intercepts and slopes were fit over the support of "years since first cognitive assessment". We included age as a population-level (fixed) effect to regularize slopes. Typically (for most cognitive tests), participants improve with practice following each assessment, and cognitive test performance tends to decline with age (1). Practise effects tend to be non-linear (reaching an asymptote) (2), so we used data from 2015 onwards to obtain a locally linear approximation during the period of interest. Hierarchical (aka 'mixed effects') models are appropriate when decomposing these competing trends, since observations are not independent. We used Bayesian hierarchical estimation because observations were not equally spaced and some observations were missing so it would not have been appropriate to use methods which required complete cases (e.g. latent growth curve models). This also gave us flexibility to model error distributions appropriate for each test, which we will discuss below.

Weakly regularizing student-t priors (df = 5) were used for the population-level age coefficients. Default priors in `brms` R package (v2.16.1) (3, 4) were used for remaining parameters. Where a Gaussian did not adequately model the residuals (e.g. right-skewed data, ceiling effects) we log-transformed scores or fitted beta-binomial models (5). A univariate model was fitted for each cognitive test. Four chains were run for 10^3^ iterations after 3x10^3^ iterations warmup, checking trace plots and R-hat statistics to confirm adequate model convergence. We used (graphical) posterior predictive checks to confirm that the observed data fell adequately within the distribution of model predictions (6).

The posterior predictive point estimate (mean) was used to infer by-participant cognitive test scores coinciding with blood collection in 2018, whether missing or not.

## Latent Variables

Five items were included in the latent variable model for episodic memory, Paired Associates Learning Test (PAL), Rey Auditory Verbal Learning Test (RAVLT), Rey Complex Figure Test (RCFT) and WMS-III Logical Memory Test I and II (LMi, LMii). We removed LMi and RAVLT because they had low correlation with other items and LMi was highly correlated with LMii, and this improved the model fit to meet criteria (Table 1 in manuscript). Four items were included in the latent variable model for executive function, Trail Making Test Part B (TMTb), WAIS-III Digit Span Test (WAIS-DS), Controlled Oral Word Association Test (COWAT), Stroop Test C (Table 1). We removed COWAT because it was poorly correlated with other items and this improved model fit to meet criteria. The first latent variable model for language did not quite meet criteria but was not improved by omitting instruments, so we included WAIS-III Vocabulary, WAIS-III Comprehension, Boston Naming Test as planned (Table 1).

**Supplementary Results**


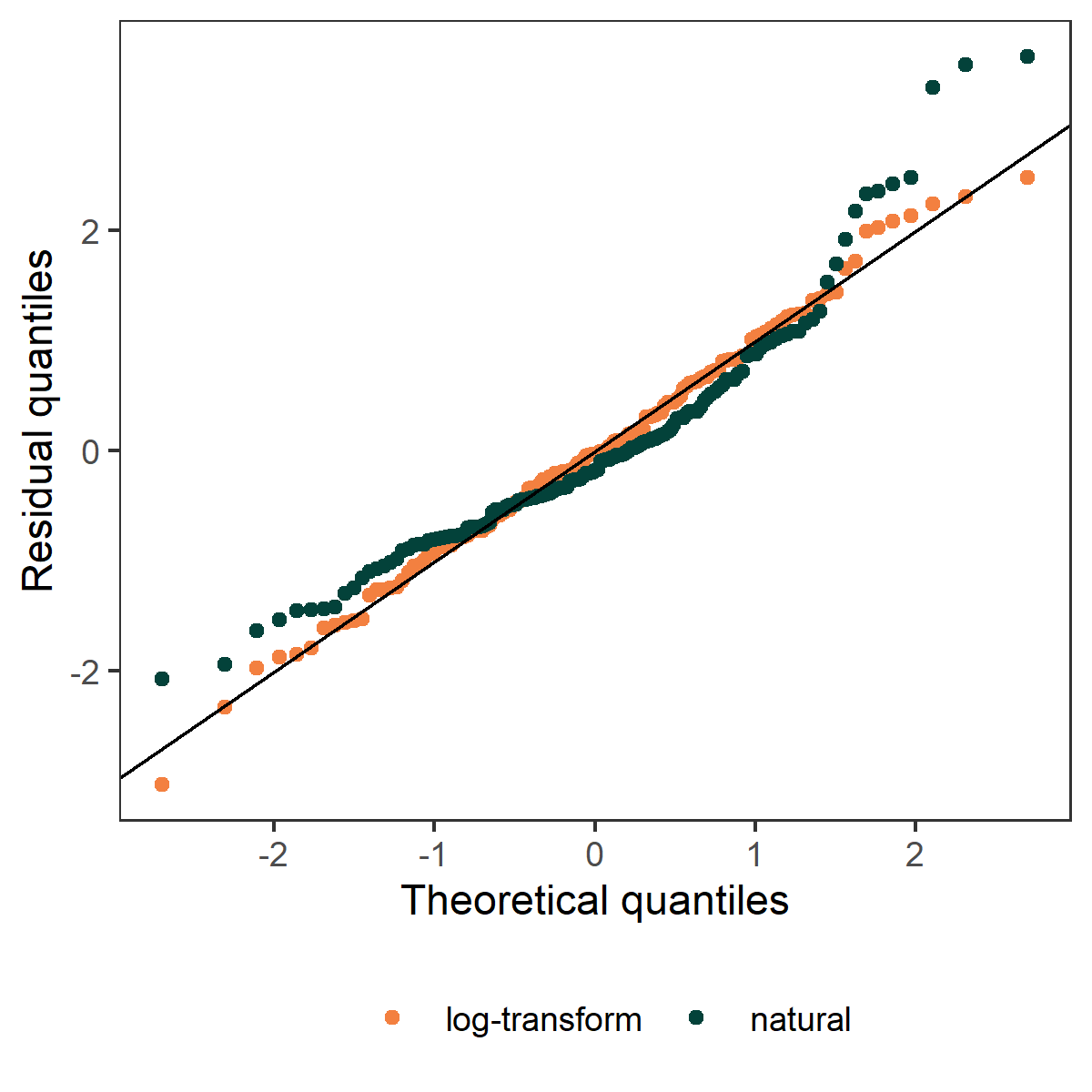


**Supplementary Figure 1.** Q-Q plot of natural (black) and log_e_-transformed (orange) serum NfL levels (pg/ml) regressed on age. This figure demonstrates that the relationship is approximately log_e_-linear, and that normality of residuals is improved with log_e_-transformation of serum NfL (pg/ml) data.

**Sensitivity analysis of time-coincident raw cognitive test scores**

To investigate whether using raw scores, rather than modelled scores changed the statistical conclusions, we performed the following sensitivity analysis by analysing only cognitive assessments which coincided with blood collections in the same calendar year (2018), a complete-case analysis.

The number of participants who had cognitive testing in the same year as blood sampling (2018) was N=75, 70.7% were females. Mean serum NfL concentration was 14.5pg/ml (SD = 7.71). The mean age of participants was 67 years (SD = 6.55). Mean years of education was 16.3 years (SD = 3.7). Latent variable models are reported in Supplementary Table 1. Results of the mediation analysis are in Supplementary Table 2. The analysis demonstrated that serum NfL levels did not mediate the relationship between age and cognitive functions across the domains of executive function, episodic memory and language. The results of this sensitivity analysis therefore support the findings reported in the Results section of the manuscript.

**Supplementary Table 1.** Cognitive domain latent variables, cognitive tests comprising them and factor loadings ± 95% CI using time coincident (2018), raw cognitive test data only.

| Cognitive Domain | Cognitive Tests in model | LV Loading | Lower CI of LV Loading | Upper CI of LV Loading | CFI | RMSEA | Model test statistic | p-value | R^2^ |
| --- | --- | --- | --- | --- | --- | --- | --- | --- | --- |
| *Executive Function | Trail Making Test Part B  Digit-span, WAIS-III  Stroop C | .667  .327  .814 | .464  -.078  .453 | .869  .732  1.176 | 1.00 | .000 | $\chi_{7}^{2}=$  6.1 | .534 | .295 |
| Episodic Memory | Paired Associates Learning  Rey Complex Figure Test  Logical Memory (I) subtest, WME | .644  .729  .553 | .003  .027  -.466 | 1.286  1.431  1.571 | .981 | .053 | $\chi_{7}^{2}=$  8.5 | .294 | .237 |
| Language | Boston naming test  Vocabulary, WAIS-III  Comprehension, WAIS-III | .654  .864  .484 | .418  .403  .003 | .890  1.325  .965 | .987 | .047 | $\chi_{7}^{2}=$  8.1 | .320 | .173 |

*Latent Variable (LV); Confidence Interval (CI); Comparative Fit Index (CFI); Root Mean Squared Error of Approximation (RMSEA); Wechsler Adult Intelligence Scale-third (WAIS-III); Wechsler Memory Scale (WME). *Using the same latent variable model as reported in Results and detailed above, an adequate solution was not found using the coincident observations, results are reported here for completeness but should be interpreted with caution.*

**Supplementary Table 2.** Mediation analysis results for cognitive domain latent variables using participants who had cognitive test data collected in the year (2018) coincident with blood sampling.

| Executive Function | | | | | |
| --- | --- | --- | --- | --- | --- |
| Predictor | **Dependent variable** | **Standardised Regression coefficient (β)** | **Lower CI of β** | **Upper CI of β** | **p-value** |
| Age | *Executive Function LV | -.613 | -.844 | -.383 | <.001 |
| Serum NfL | *Executive Function LV | .135 | -.113 | .383 | .333 |
| Education | *Executive Function LV | .025 | -.192 | .242 | .820 |
| Age | Serum NfL | .610 | .504 | .716 | <.001 |
| a*b | Indirect Pathway | .083 | -.072 | .237 | .296 |
| a*b/(c+a*b) | Proportion Mediated | .156 | -.145 | .456 | .311 |
| Episodic Memory | | | | | |
| Predictor | **Dependent variable** | **Standardised Regression coefficient (β)** | **Lower CI of β** | **Upper CI of β** | **p-value** |
| Age | Episodic Memory LV | -.588 | -1.216 | .040 | .066 |
| Serum NfL | Episodic Memory LV | .275 | -.131 | .681 | .184 |
| Education | Episodic Memory LV | .094 | -.238 | .426 | .579 |
| Age | Serum NfL | .610 | .507 | .713 | <.001 |
| a*b | Indirect Pathway | .168 | -.085 | .420 | .194 |
| a*b/(c+a*b) | Proportion Mediated | -.399 | -1.000 | .203 | .194 |
| Language | | | | | |
| Predictor | **Dependent variable** | **Standardised Regression coefficient (β)** | **Lower CI of β** | **Upper CI of β** | **p-value** |
| Age | Language LV | -.295 | -.569 | -.022 | .034 |
| Serum NfL | Language LV | -.002 | -.346 | .341 | .990 |
| Education | Language LV | .271 | -.019 | .562 | .067 |
| Age | Serum NfL | .650 | .535 | .765 | <.001 |
| a*b | Indirect Pathway | -.013 | -.225 | .222 | .990 |
| a*b/(c+a*b) | Proportion Mediated | .005 | -.745 | .755 | .990 |

*Latent Variable (LV); Standardised Regression coefficient (β); Confidence Interval (CI); Neurofilament Light (NfL). *Using the same latent variable model as reported in Results and detailed above, an adequate solution was not found using the coincident observations, results are reported here for completeness but should be interpreted with caution.*

**Interaction analyses**

To account for possible confounding and moderation of results due to sex and APOE genotype we conducted further analysis to supplement the reported Results. Cognitive domain latent variable scores were projected for each participant using the latent variable models used for the main analysis. We then regressed these scores on APOE genotype x log_e_-transformed serum NfL, APOE genotype x age and sex x log_e_-transformed serum NfL interaction terms, adjusted for education. We have reported results of an F-test on model parameters using type 3 sums of squares. Results are presented below in Supplementary Table 3 for APOE genotype x log_e_-transformed serum NfL, Supplementary Table 4 for APOE genotype x age and Supplementary Table 5 for sex x log_e_-transformed serum NfL. None of the interactions were significant.

**Supplementary Table 3.** F-test results and model parameters for the relationship between cognitive domain latent variables and log_e_-transformed serum NfL levels, with the inclusion of a log_e_-transformed serum NfL x APOE genotype interaction.

| Executive Function | | | | |
| --- | --- | --- | --- | --- |
| Effect | **Df** | **SS** | **F-value** | **p-value** |
| Age | 1 | 50.779 | 208.956 | <.001 |
| Serum NfL | 1 | .033 | .136 | .713 |
| APOE genotype | 1 | .003 | .012 | .912 |
| Education | 1 | 4.359 | 17.937 | <.001 |
| Serum NfL x APOE genotype | 1 | .066 | .272 | .603 |
| Residuals | 127 | .243 |  | |
| Episodic Memory | | | | |
| Effect | **Df** | **SS** | **F-value** | **p-value** |
| Age | 1 | 22.579 | 84.107 | <.001 |
| Log_e_ NfL | 1 | .019 | .071 | .790 |
| APOE genotype | 1 | .001 | .004 | .947 |
| Education | 1 | 1.960 | 7.301 | .008 |
| Serum NfL x APOE genotype | 1 | .0032 | .012 | .914 |
| Residuals | 127 | 34.094 |  | |
| Language | | | | |
| Effect | **Df** | **SS** | **F-value** | **p-value** |
| Age | 1 | 2.831 | 26.724 | <.001 |
| Log_e_ NfL | 1 | .024 | .222 | .638 |
| APOE genotype | 1 | .013 | .123 | .727 |
| Education | 1 | 7.462 | 70.448 | <.001 |
| Serum NfL x APOE genotype | 1 | .103 | .969 | .327 |
| Residuals | 127 | .106 |  | |

*Degrees of freedom (DF); Sum of Squares (SS); Neurofilament Light (NfL); Apolipoprotein (APOE).*

**Supplementary Table 4.** F-test results and model parameters for the relationship between cognitive domain latent variables and log_e_-transformed serum NfL levels, with the inclusion of a APOE genotype x age interaction.

| Executive Function | | | | |
| --- | --- | --- | --- | --- |
| Effect | **Df** | **SS** | **F-value** | **p-value** |
| Age | 1 | 50.779 | 208.858 | <.001 |
| APOE genotype | 1 | .003 | .012 | .912 |
| Serum NfL | 1 | .033 | .136 | .713 |
| Education | 1 | 4.359 | 17.929 | <.001 |
| Age x APOE genotype | 1 | .052 | .212 | .646 |
| Residuals | 127 | .243 |  | |
| Episodic Memory | | | | |
| Effect | **Df** | **SS** | **F-value** | **p-value** |
| Age | 1 | 22.579 | 85.174 | <.001 |
| APOE genotype | 1 | .001 | .004 | .948 |
| Serum NfL | 1 | .019 | .072 | .789 |
| Education | 1 | 1.960 | 7.394 | .007 |
| Age x APOE genotype | 1 | .430 | 1.622 | .205 |
| Residuals | 127 | .265 |  | |
| Language | | | | |
| Effect | **Df** | **SS** | **F-value** | **p-value** |
| Age | 1 | 2.831 | 26.526 | <.001 |
| APOE genotype | 1 | .013 | .122 | .727 |
| Serum NfL | 1 | .024 | .220 | .640 |
| Education | 1 | 7.462 | 69.926 | <.001 |
| Age x APOE genotype | 1 | .002 | .021 | .884 |
| Residuals | 127 | .107 |  | |

*Degrees of freedom (DF); Sum of Squares (SS); Neurofilament Light (NfL); Apolipoprotein (APOE).*

**Supplementary Table 5.** F-test results and model parameters for the relationship between cognitive domain latent variables and log_e_-transformed serum NfL levels, with the inclusion of a sex x log_e_-transformed serum NfL.

| Executive Function | | | | |
| --- | --- | --- | --- | --- |
| Effect | **Df** | **SS** | **F-value** | **p-value** |
| Age | 1 | 54.366 | 225.294 | <.001 |
| Serum NfL | 1 | .011 | .044 | .834 |
| Sex | 1 | .128 | .529 | .468 |
| Education | 1 | 4.777 | 19.798 | <.001 |
| Serum NfL x Sex | 1 | .401 | 1.661 | .200 |
| Residuals | 137 | 33.060 |  | |
| Episodic Memory | | | | |
| Effect | **Df** | **SS** | **F-value** | **p-value** |
| Age | 1 | 23.272 | 92.453 | <.001 |
| Serum NfL | 1 | .017 | .068 | .794 |
| Sex | 1 | .157 | .622 | .432 |
| Education | 1 | 2.149 | 8.536 | .004 |
| Serum NfL x Sex | 1 | .247 | .980 | .324 |
| Residuals | 137 | 34.485 |  | |
| Language | | | | |
| Effect | **Df** | **SS** | **F-value** | **p-value** |
| Age | 1 | 22.579 | 84.107 | <.001 |
| Serum NfL | 1 | .019 | .071 | .790 |
| Sex | 1 | .001 | .004 | .947 |
| Education | 1 | 1.960 | 7.301 | .008 |
| Serum NfL x Sex | 1 | .003 | .012 | .914 |
| Residuals | 137 | 34.094 |  | |

*Degrees of freedom (DF); Sum of Squares (SS); Neurofilament Light (NfL); Apolipoprotein (APOE).*

**References**

1. Bindoff AD, Summers MJ, Hill E, Alty J, Vickers JC. Studying at university in later life slows cognitive decline: A long-term prospective study. Alzheimers Dement (N Y). 2021;7(1):e12207. doi:10.1002/trc2.12207

2. Alty JE, Bindoff AD, Stuart KE, Roccati E, Collins JM, King AE, et al. Sex-Specific Protective Effects of Cognitive Reserve on Age-Related Cognitive Decline: A 5-Year Prospective Cohort Study. Neurology. 2023;100(2):e211-e9. doi:10.1212/WNL.0000000000201369

3. Burkner PC. brms: An R Package for Bayesian Multilevel Models Using Stan. J Stat Softw. 2017;80(1):1-28. doi:10.18637/jss.v080.i01

4. Team RC. R: A languageand environment for statistical computing.: R Foundation for Statistical Computing, Vienna, Austria.; 2021 [Available from: <https://www.R-project.org/>.

5. Muniz-Terrera G, van den Hout A, Rigby RA, Stasinopoulos DM. Analysing cognitive test data: Distributions and non-parametric random effects. Stat Methods Med Res. 2016;25(2):741-53. doi:10.1177/0962280212465500

6. Schad DJ, Betancourt M, Vasishth S. Toward a principled Bayesian workflow in cognitive science. Psychol Methods. 2021;26(1):103-26. doi:10.1037/met0000275
